# Chitosan–Aspirin Combination Inhibits Quorum-Sensing Synthases (*lasI* and *rhlI*) in *Pseudomonas aeruginosa*

**DOI:** 10.3390/life14040481

**Published:** 2024-04-05

**Authors:** Mona Shaban E. M. Badawy, Omnia Karem M. Riad, Marwa F. Harras, Reem Binsuwaidan, Asmaa Saleh, Samar A. Zaki

**Affiliations:** 1 Department of Microbiology and Immunology, Faculty of Pharmacy (Girls), Al-Azhar University, Cairo 11651, Egypt; samarzaki@azhar.edu.eg; 2 Pharmaceutical Medicinal Chemistry Department, Faculty of Pharmacy (Girls), Al-Azhar University, Cairo 11651, Egypt; marwaharras.pharmg@azhar.edu.eg; 3 Department of Pharmaceutical Sciences, College of Pharmacy, Princess Nourah bint Abdulrahman University, P.O. Box 84428, Riyadh 11671, Saudi Arabia; rabinsuwaidan@pnu.edu.sa (R.B.); asali@pnu.edu.sa (A.S.)

**Keywords:** quorum sensing, *Pseudomonas aeruginosa*, antimicrobials, resistance, *lasI*, *rhlI*

## Abstract

Background: Quorum sensing (QS) controls the virulence of *P. aeruginosa.* This study aims to determine the anti-QS activity of aspirin alone and in combination with chitosan to reach maximum inhibition. We tested ten virulent *Pseudomonas aeruginosa* (*P*. *aeruginosa*) isolates and screened for N-acyl homoserine lactone (AHL) production using *Agrobacterium tumefaciens* as a biosensor. *P. aeruginosa* isolates were treated with sub-minimum inhibitory concentrations (MICs) of aspirin and chitosan–aspirin. We used broth microdilution and checkerboard titration methods to determine the MICs and the synergistic effect of these two compounds, respectively. Real-time polymerase chain reaction (PCR) was used to estimate the anti-QS activity of the aspirin–chitosan combination on the expression of *lasI* and *rhlI* genes. Results: Aspirin decreased the motility and production of AHLs, pyocyanin, and biofilm. Chitosan potentiated the inhibitory effect of aspirin. The chitosan–aspirin combination inhibited *lasI* and *rhlI* gene expression in PAO1 (ATCC 15692) by 7.12- and 0.92-fold, respectively. In clinical isolates, the expression of *lasI* and *rhlI* was decreased by 1.76 × 10^2^- and 1.63 × 10^4^-fold, respectively. Molecular docking analysis revealed that aspirin could fit into the active sites of the QS synthases *lasI* and *rhlI* with a high binding affinity, causing conformational changes that resulted in their inhibition. Conclusions: The chitosan–aspirin combination provides new insights into treating virulent and resistant *P. aeruginosa.*

## 1. Background

*P. aeruginosa* is a pathogenic Gram-negative bacterium found in hospitals, especially in burn units and implanted medical devices [1]. It can survive under drastic conditions as it has many virulence factors regulated by QS [2].

The main QS systems of *Pseudomonas aeruginosa* are dependent on the two-component systems (TCSs) *lasI*/*lasR*, *rhlI*/*rhlR*, PqsABCDE/PqsR (MvfR), and AmbBCDE/IqsR. These TCSs participate in the collective reaction to extracellular signaling autoinducer molecules, including N-(3-oxododecanoyl) homoserine lactone (3-Oxo-C12-HSL), encoded by *lasI*; N-butyryl-L-homoserine lactone (C4-HSL), encoded by *rhlI*; 2-heptyl-3-hydroxy-4-quinolone (PQS), encoded by PqsABCD and PqsH; and 2-(2-hydroxyphenyl)-thiazole-4-carbaldehyde (IQS), encoded by AmbBCDE [3]. The autoinducer 3OH-C12-HSL activates the transcription factor *lasR* in the las system, which subsequently drives the expression of *lasI*, which initiates the synthesis of exotoxin A, LasA protease, and LasB elastase. By binding to and interacting with rhlR in the rhl TCS, the autoinducer C4-HSL increases RhlI expression and triggers the controlled synthesis of cytotoxic lectins, rhamnolipids, lasB elastase, and pyocyanin, all of which are essential for virulence and the formation and development of biofilms. The QS systems Las, Rhl, Pqs, and Iqs are interconnected hierarchically [4,5].

Since the QS mechanism and its critical role in bacterial virulence were discovered, novel targets have been identified for reducing bacterial pathogenicity. QS can be disrupted using natural microbial products, which are integral for developing novel therapeutic chemicals [6]. The continuous emergence of multidrug-resistant (MDR) pathogens necessitates using QS inhibition methods instead of bactericidal and bacteriostatic techniques to avoid virulence [7].

As several clinically relevant bacteria become increasingly resistant to commonly used antimicrobial agents, the focus has shifted to developing novel antimicrobial therapeutics from different pharmacological classes. Using drugs with varying properties, physiological activities, and functions might benefit different areas of medicine. This has led to the realization that drugs from other pharmacological classes might exhibit possible antimicrobial activity [8]. Non-steroidal anti-inflammatory drugs (NSAIDs) have shown potent antimicrobial activity against several Gram-positive and Gram-negative bacteria, with MICs ranging between 50 and 200 g mL^−1^ and even lower in some cases [9].

Even though aspirin is mainly used to treat pain, it exhibits broad-spectrum antimicrobial activity against some planktonic and biofilm cultures [10]. El-Mowafy et al. [11] demonstrated that aspirin could effectively inhibit QS, virulence, and toxins in *P. aeruginosa.* Moreover, chitosan displayed anti-QS activity by inhibiting the *P. aeruginosa lasI* and *rhlI* genes, which control QS [12,13].

This study aims to examine the effect of an aspirin–chitosan combination on the virulence factors of *P. aeruginosa*, which are controlled by QS (including motility, pyocyanin production, and biofilm formation), and on the expression of the *lasI* and *rhlI* genes. We also investigated the structural basis of aspirin’s inhibitory activity. We applied molecular docking to explore the possible interactions and the binding pattern of aspirin to the *lasI* and *rhlI* active sites.

## 2. Methods

We selected 10 clinical *P. aeruginosa* isolates, which were previously screened from 100 isolates based on motility, QS activity, biofilm, and pyocyanin production [13]. Their antimicrobial susceptibility pattern, motility, biofilm formation, pyocyanin, and acyl homoserine production, along with the minimum inhibitory concentration (MIC) of chitosan, were also previously reported [13]. We evaluated the effects of aspirin and the aspirin–chitosan combination on these isolates.

### 2.1. Bacterial Strains and Media

We used the standard *P. aeruginosa* strain PAO1 (ATCC 15692) and the ten previously studied clinical isolates [13]. From frozen culture stocks (−80 °C), they were sub-cultured in a nutrient-rich Luria–Bertani broth containing 1% peptone, 0.5% yeast extract, and 1% sodium chloride (pH 7.4) at 37 °C for 24 h, with shaking [13]. The motility of the *P. aeruginosa* isolates was tested using swimming [14] and swarming media [15]. A pyocyanin assay was conducted using King A broth medium [16].

*A. tumefaciens* KYC55 (pJZ372; pJZ384; pJZ410) was used as a biosensor for the AHLs. *A. tumefaciens* was cultured in AT limited salt medium supplemented with 100 g mL^−1^ spectinomycin, 100 g mL^−1^ gentamicin, and 4 g mL^−1^ tetracycline to preserve the required plasmids [17]. The antibiotics and chemicals were purchased from Sigma-Aldrich, Germany.

### 2.2. Inhibitory Activity of Aspirin and Chitosan–Aspirin against P. aeruginosa Virulence Factors

#### 2.2.1. Determination of MICs of Aspirin and Chitosan–Aspirin

The MICs of aspirin (10 mg/mL) and chitosan (26.666 mg/mL) were determined using broth microdilution, as recommended by CLSI [18]. The preparation of aspirin and chitosan and the determination of the MICs and sub-MICs were performed based on [13,18]. In brief, the tested agents were used for preparing 2-fold serial dilutions in MHB. Diluted tested agents were inoculated with 5 μL *P. aeruginosa* culture, equivalent to a 0.5 McFarland standard, and incubated at 37 °C for 24 h. The MIC was calculated as the lowest concentration of tested agents that inhibited the visible growth of the organism.

Chitosan (26.666 mg/mL) was mixed with aspirin (10 mg/mL) to determine the synergism between the two compounds using an interaction (synergy) study based on the checkerboard titration method. For antimicrobial combinations, the fractional inhibitory concentration (FIC) index was determined using the following equation:

FIC index=FICA+FICB=A/MICA+B/MICB

where *MICA* and *MICB* are the MICs of chitosan and aspirin alone, and *A* and *B* are the MICs of the chitosan–aspirin in combination [19].

#### 2.2.2. Phenotypic Identification of Aspirin and Chitosan–Aspirin Inhibitory Activity

The isolates were treated with the sub-MICs of aspirin and chitosan–aspirin, as previously described by Badawy et al. [13], to observe their effect on *P. aeruginosa* virulence factors (QS activity, motility, biofilm, and pyocyanin production).

#### 2.2.3. Genotypic Identification of Chitosan–Aspirin Inhibitory Activity

RNA was extracted from the *P. aeruginosa* clinical isolates and PAO1 using Gene JET RNA Purification Kit (Thermo Scientific, Lithuania, Vilnius, Caracas, Venezuela) according to the manufacturer’s guidelines. Thermo Scientific Verso SYBR Green 1-Step QRT-PCR and ROX Vial (Thermo Scientific, Lithuania) kits were used to synthesize the complementary DNA from the RNA. Real-time PCR was used to evaluate the effect of sub-MICs of chitosan–aspirin on the QS genes (*lasI and rhlI*) in the treated and untreated cultures using the primers for *lasI*, *rhlI*, and *ropD*. All experiments were performed in duplicates. *rop*D is the used reference genes to normalize the results in real time PCR. The following primers were used for the amplification of *rhlI* forward 5′-GTAGCGGGTTTGCGGATG-3′ and *rhlI* reverse 5′-CGGCATCAGGTCTTCATCG-3′; *lasI* forward, 5′-CGCACATCTGGGAACTCA-3′ and *lasI* reverse 5′-CGGCACGGATCATCATCT-3′; *ropD* forward, 5′-CGAACTGCTTGCCGACTT-3′ and *ropD* reverse, 5′-GCGAGAGCCTCAAGGATAC-3′ [20]. The primer sequences were checked using the Basic Local Alignment Search Tool (BLAST) (NCBI).

### 2.3. Homology Modeling

The *rhlI* synthase domain sequence, obtained from the UniProtKB database (www.uniprot.org, entry P5429, accessed on 2 March 2022), was utilized to search for the templates with high identity from the Protein Data Bank (PDB) database. The best-matched template (PDB ID: 3P2H) with 37.99% sequence identity was selected to build the homology model using the Swiss model (www.swissmodel.expasy.org/ accessed on 4 March 2022). ModRefiner was used for protein refinement and energy minimization.

#### 2.3.1. Homology Model Validation

The generated 3D model of the *rhlI* synthase was validated using PROCHECK to calculate the Ramachandran plot and ERRAT scores to investigate the energy criteria of this model with the template structure.

#### 2.3.2. Molecular Docking

We performed semi-flexible molecular docking using Vina Autodock V1.5.7, freely available software. The crystal structures of *lasI* were retrieved from the PDB (PDB ID: 1RO5), while the *rhlI* 3D structure was built using homology modeling. For docking using Vina Autodock, the ligand and receptor were adjusted in PDBQT format. Additionally, the coordinates and dimensions of the grid box around the binding sites were determined using M.G.L tools, and the docking simulation was conducted. The molecular docking results were visualized using the Biovia discovery-studio 2020 visualizer to generate the predicted interactions in 2D and 3D modes for aspirin (https://3dsbiovia.com/resource-center/downloads/ accessed on 20 April 2022).

### 2.4. Statistical Analyses

IBM SPSS^®^ Statistics version 22 (IBM^®^ Corp., Armonk, NY, USA) was used for the statistical analyses. The values were expressed as the mean and standard deviation (SD) or median and range, whichever was applicable. Frequency and percentage were used to express the qualitative data.

## 3. Results

The highly virulent *P. aeruginosa* isolates (*n* = 10) displayed motility, QS, biofilm formation, and pyocyanin production, as previously mentioned [13]. The antibiogram of those virulent isolates proved that 30% were MDR while 70% were non-MDR.

### 3.1. Inhibitory Activity of Aspirin and Chitosan–Aspirin against P. aeruginosa Virulence Factors

#### 3.1.1. MICs of Aspirin and Chitosan–Aspirin

The MIC values of aspirin and chitosan–aspirin ranged from 0.156 to 2.5 and 0.035 to 0.143 mg mL^−1^, respectively. The results of interaction studies between the chitosan and aspirin revealed the presence of synergism between them (Table 1). The FIC indexes were interpreted as follows: ≤0.5, synergy; 0.5–4.0, neutral; and >4.0, antagonism.

#### 3.1.2. Phenotypic Identification of Aspirin and Chitosan–Aspirin’s Inhibitory Activity

Aspirin reduced AHL, pyocyanin, and biofilm formation in PAO1 and the virulent clinical isolates (Table 2). Biofilm formation was also inhibited in the clinical isolates, and the optical density (OD) of the mean (±SD) was reduced from 0.240 to 0.075 to 0.012 to 0.005 after treatment with the sub-MIC of aspirin (Table 2). Aspirin decreased the production of pyocyanin in PAO1 from 19.88 to 11.41 µg mL^−1^. It also decreased the mean (±SD) pyocyanin production from 21.96 (±8.19) to 12.24 (±2.623) µg mL^−1^ in the clinical isolates (Table 2).

**Figure 1:**
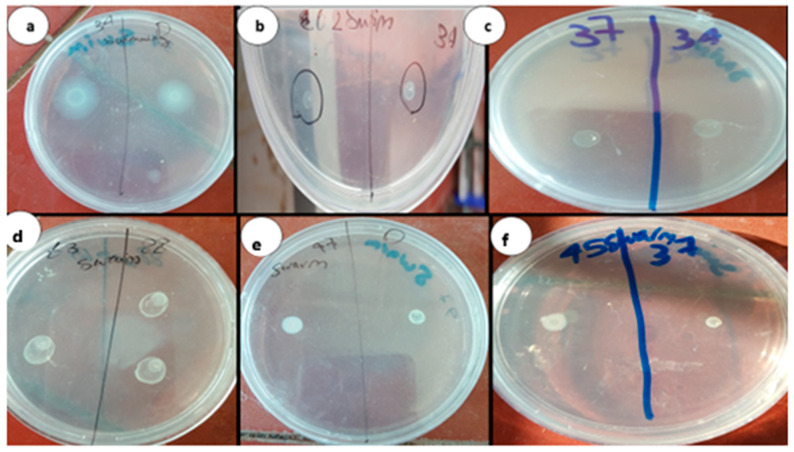
Swimming and swarming motility assay. Swimming and swarming motility were inhibited by aspirin (**b**,**f**) and chitosan–aspirin (**c**,**d**), respectively; (**a**,**e**) represent the swimming and swarming motility controls, respectively.

Aspirin significantly decreased AHL production in all the strains. Swimming and swarming motility were also reduced in all isolates after treatment with the sub-MIC of aspirin (Table 2, Figure 1).

Combining chitosan with aspirin significantly increased the chitosan’s inhibitory activity (*p*-value = 0.01). We observed a substantial drop in AHL production as the mean zone diameters of the clinical isolates decreased from 2 (±0) to 0.4 (±0.5). The concentration of pyocyanin produced by the standard strain PAO1 was decreased from 19.888 to 6.196, and the mean of pyocyanin production by the clinical isolates was decreased from 21.963 (±8.192) to 6.353 (±01.337). Aspirin–chitosan decreased biofilm development (the OD of PAO1 was reduced to 0.01, mean ODs of the clinical isolates were reduced from 0.24 to 0.010 (±0.001)). Motility, i.e., swimming, and swarming activities were reduced from 5.9 (±0.7) and 5.6 (±1.0) to 2.9 (±0.3) and 2.3 (±0.5) mm, respectively (Table 2).

#### 3.1.3. Genotypic Identification of Chitosan–Aspirin’s Inhibitory Activity

The chitosan–aspirin combination significantly increased chitosan’s inhibitory activity at the genotypic level (*p-*value = 0.01). In several virulent isolates, chitosan–aspirin induced a greater decrease in the expression of *lasI* and *rhlI* genes than chitosan alone, with transcription of *lasI* and *rhlI* being decreased by 1.76 × 10^2^- and 1.63 × 10^4^-fold, respectively. After treatment with chitosan–aspirin, the values ranged from 0.47 to 3.95 × 10^9^ in *lasI* and from 1.73 to 1.83 × 10^7^ in *rhlI*. Table 3 shows that the *lasI* expression in PAO1 was reduced by 7.12-fold after treatment with chitosan–aspirin.

### 3.2. Molecular Docking Study

**Figure 2:**
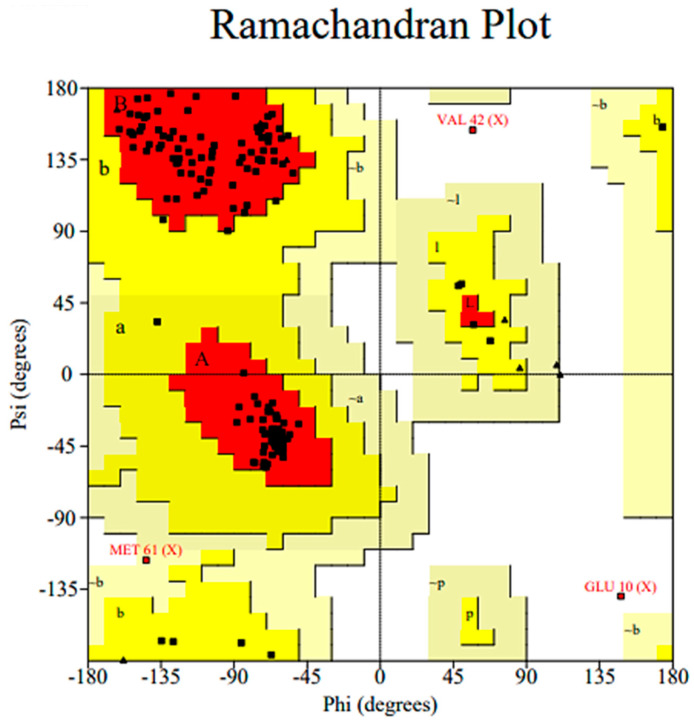
Ramachandran plot of the *rhlI* 3D model. The most favored regions are colored red, additional allowed are colored yellow, generously allowed are colored light yellow and disallowed regions are indicated as white fields.VAL: valine. GLU: Glutamic acid, MET: Methionine.

The 3D crystal structures of *lasI* (PDB ID: 1RO5) were obtained from the PDB [21]. The 3D structure of *rhlI* synthase was modeled using the SWISS MODEL homology method, as it was not available in the PDB. Then, energy minimization and refinement of the modeled structure were performed using ModRefiner. Ramachandran plot analysis was used to validate the final model based on the chi (Φ) and psi (Ψ) values. Figure 2 shows the Ramachandran plot of the modeled *rhlI*, with 90.7% of residues in the preferred region, 8.5% in the permitted regions, and 0.8% in the outlier region. We validated the model using ERRAT, a verification algorithm for protein structure that can differentiate between correctly and incorrectly determined areas of protein structures depending on specific atomic interactions [22]. It provides an “overall quality factor” value, the percentage of proteins with an error value less than the statistical rejection limit of 95%. The ERRAT scores of the model and template were 97.5 and 95.9, respectively, indicating that the model is high-quality and suitable for molecular docking.

**Figure 3:**
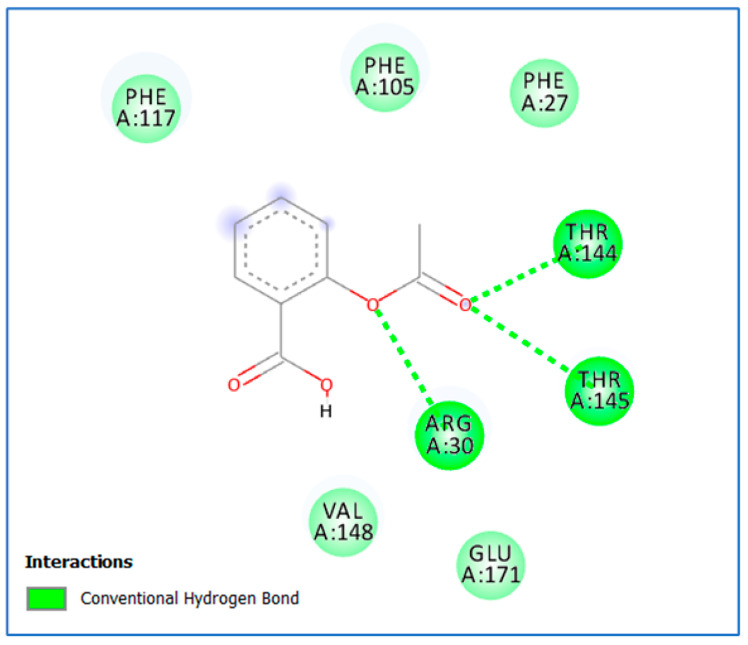
Aspirin’s proposed 2D binding mode within the *lasI* active site.

**Figure 4:**
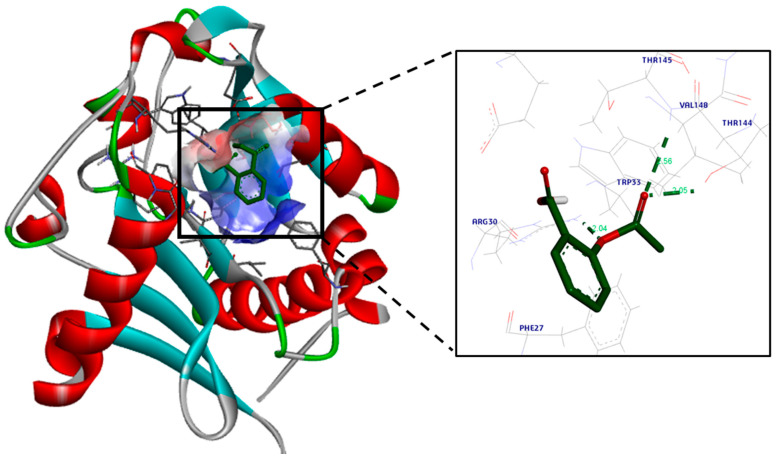
Aspirin’s proposed 3D binding mode within the *lasI* active site. Hydrogen bonds are displayed as green dotted lines, with the bond length in Å. Oxygen atoms are colored red and hydrogen atoms are colored white. The protein is colored according to its secondary structure; Alpha helices are colored red, beta sheets are colored cyan, turns are colored grey, and all other residues are colored green.

The docking simulations for aspirin revealed a high binding affinity to *lasI*, as it fits properly in its active site cavity with a docking score of −8.8 kcal/mol (Figure 3 and Figure 4). A hydrogen bond was observed between aspirin’s ester oxygen and Arg30 (2.04 Å). Moreover, the ester carbonyl group formed two hydrogen bond interactions: one formed with the important and highly conserved amino acid residue Thr144 (2.05 Å), and the second formed with Arg145 (2.56 Å).

**Figure 5:**
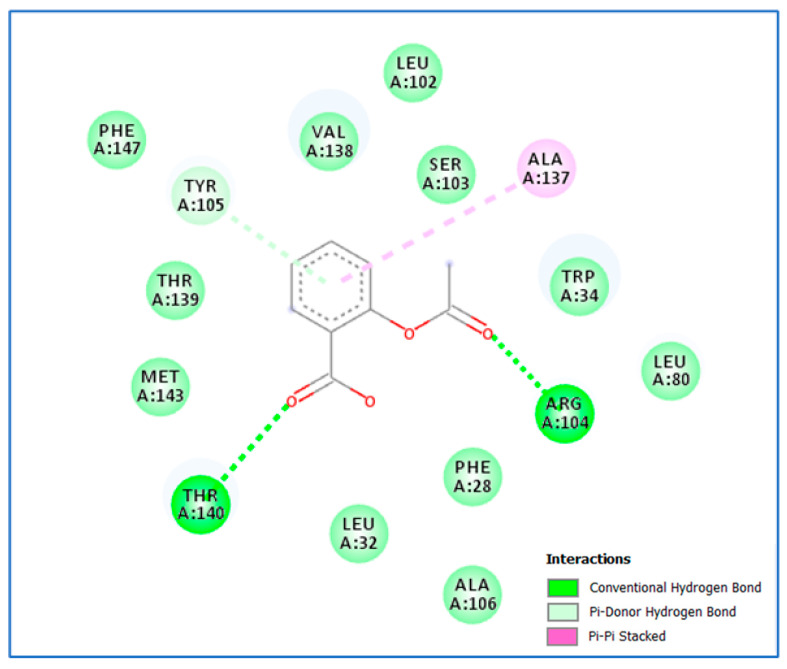
Aspirin’s proposed 2D binding mode within the *rhlI* active site.

**Figure 6:**
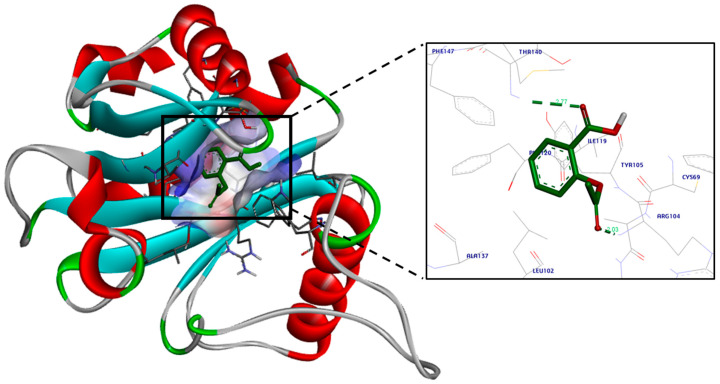
Aspirin’s proposed 3D binding mode within the *rhlI* active site. Hydrogen bonds are displayed as green dotted lines, with the bond length in Å. Oxygen atoms are colored red and hydrogen atoms are colored white. The protein is colored according to its secondary structure; Alpha helices are colored red, beta sheets are colored cyan, turns are colored grey, and all other residues are colored green.

Further, the docking study analysis of aspirin in the active site of *rhlI* displayed proper fitting with a docking score of −9.7 kcal/mol. The carboxylic acid functional group of aspirin was involved in hydrogen bond interaction with Thr140 (2.77 Å), while the ester group formed a hydrogen bond with Arg104 (2.03 Å). Furthermore, the phenyl moiety was near Tyr105, forming a Van der Waals interaction and pi–pi stacking interaction with Ala137 (Figure 5 and Figure 6).

## 4. Discussion

Research indicates that antimicrobial resistance (AMR) results in over 2.8 million documented cases and over 35,000 fatalities in the US alone each year. Despite the increasing research studying drug resistance mechanisms to find different alternative strategies [23,24,25,26,27,28,29,30], the bacterial community is well interconnected to fight any new drugs. One of the new strategies is reducing bacterial virulence and disrupting their communication by interrupting their QS [27]. Since QS is essential for *P. aeruginosa* and other pathogenic bacteria’s virulence and survival, it is a novel target for anti-infective drugs [31]. QS inhibitors do not influence bacterial growth or viability and hence do not exert heavy selective pressure, leading to resistance, unlike antibiotics. There is an urgent need to discover safe, broad-spectrum, and stable anti-QS compounds with confirmed therapeutic properties [32].

The anti-QS strategy is helpful for serious microorganisms such as *P. aeruginosa*, which has normal drug resistance and virulence mechanisms that enable its survival in harsh environments [33]. As QS has been shown to use signal molecules to coordinate various functions between bacteria [20], targeting these molecules might prove beneficial.

In the current study, we focused on finding anti-virulence and anti-QS compounds as novel strategies for combating *P. aeruginosa* by reducing their virulence by interrupting their QS. We chose to use chitosan as a promising natural biopolymer because of its special qualities, including its innate antimicrobial qualities, natural abundance, adaptability, non-toxicity, and biodegradability [34]. We combined its use with aspirin with its assumed antimicrobial properties to maximize the antibacterial efficacy [35]. We examined their effect phenotypically by detecting the AHL production using *A. tumefaciens* as a biosensor, and analyzing different virulence factors, e.g., motility, pyocyanin production, and biofilm formation. We discussed this combination genotypically by determining its effect on the synthase enzyme genes *lasI* and *rhlI* with their important role in the regulation of the QS system in *P. aeruginosa,* as they initiate the Las and Rhl pathways, and generate the signaling molecules acyl homoserine lactones (AHLs) [36].

Chitosan alone is proven to have antimicrobial, anti-virulence, and anti-Qs properties [13,37]. It is observed that aspirin can enhance the effect of other antibacterial drugs by increasing bacterial susceptibility to different antibiotics [38,39]. In the current study, we demonstrated that chitosan’s activity is potentiated by its combination with aspirin. Aspirin reduces bacterial virulence by suppressing swimming and swarming motility, pyocyanin production, and biofilm development due to its quorum-quenching effect rather than its bacteriostatic or bactericidal effects. These properties are in accordance with other studies [11,40,41].

We observed that aspirin significantly decreases AHL production in *P. aeruginosa*, consistent with the results observed by El-Mowafy et al. [11]. At the genotypic level, we examined the effect on the synthase genes *lasI* and *rhlI*, which have a key role in the interconnected QS system controlling virulence and biofilm formation in *P. aeruginosa*. The combination of chitosan and aspirin significantly increased (*p*-value = 0.01) chitosan’s inhibitory activity on gene expression. Chitosan–aspirin decreased the expression of the *lasI* and *rhlI* genes to a greater extent than chitosan alone, with the transcription of *lasI* and *rhlI* being decreased by 1.76 × 10^2^- and 1.63 × 10^4^- fold, respectively. However, the inhibitory effect on different isolates showed high variability, and hence the inhibition activity was represented as a median rather than a mean.

El-Mowafy et al. [11] found that aspirin suppresses the expression of QS-regulatory genes (*lasI* and *rhlI*) at the transcriptional level. *lasI* expression was inhibited by aspirin by 38%. Moreover, aspirin reduced the expression of the C4-HSL synthase gene *rhlI* by 72%. The 2^−∆∆Ct^ approach was used to determine the QS-regulated genes’ relative expression levels. Aspirin inhibited the expression of *lasI* and *rhlI* by 38% and 69%, respectively, at sub-MIC levels.

To know more about the effect of aspirin as an anti-QS molecule at the molecular level, we performed a molecular docking study to elucidate the possible binding pattern of aspirin to the active sites of both *lasI* and *rhlI*, and we predicted its affinity to the prospective targets using a scoring function based on the interactions and binding energy. We showed that the aspirin molecule fits inside the active sites of *lasI* and *rhlI*, which might result in conformational changes to these proteins and their subsequent suppression. This is consistent with the previous related study.

To our knowledge, this paper is the first report on the impact of a chitosan–aspirin combination on the inhibition of *P. aeruginosa* QS-dependent virulence factors, as no related studies have been published so far. This paper may shed a light on drug repurposing of the currently available molecules as anti-QS agents to use them in the fight against bacterial resistance.

## 5. Conclusions

Novel applications of already-available approved drugs are recommended to control antibiotic-resistant bacteria, such as *P. aeruginosa*. Aspirin, a commonly used NSAID, inhibited QS in *P. aeruginosa,* and this inhibitory function was enhanced by chitosan. The chitosan–aspirin combination inhibited AHL development, biofilm formation, pyocyanin production, motility, and the *lasI* and *rhlI* synthases. Furthermore, the molecular docking study showed that aspirin fits into the *lasI* and *rhlI* active sites in the correct orientation, rationalizing its potent inhibitory activity.

## Figures and Tables

**Table 1:** Interaction studies between chitosan and aspirin.

Isolate No.	MIC of Aspirin	MIC of Chitosan	MIC of Aspirin in Combination	MIC of Chitosan in Combination	MIC of Ch.–Asp.	FIC Index	Interpretation
ps34	0.625	0.052	0.156	0.013	0.071	0.499	Synergism
ps36	2.5	0.416	0.3125	0.052	0.071	0.25	Synergism
ps37	1.25	0.416	0.156	0.104	0.017	0.37	Synergism
ps45	1.25	1.666	0.3125	0.208	0.071	0.37	Synergism
ps47	1.25	0.208	0.156	0.026	0.035	0.24	Synergism
ps62	0.625	0.104	0.07	0.013	0.035	0.23	Synergism
ps63	1.25	0.104	0.3125	0.026	0.143	0.5	Synergism
ps88	1.25	0.104	0.156	0.013	0.143	0.24	Synergism
ps99	1.25	0.104	0.156	0.013	0.143	0.24	Synergism
ps100	1.25	0.208	0.3125	0.013	0.071	0.31	Synergism
PAO1	0.156	0.052	0.07	0.0065	0.071	0.5	Synergism

**Table 2:** Effects of aspirin and chitosan–aspirin on *P. aeruginosa* virulence factors.

		Swimming Motility, 24 h. (mm)				Swarming Motility, 24 h. (mm)				Biofilm Formation (Å)				Pyocyanin (µg mL^−1^)				QS (Score)		
	C	Ch	Asp.	Ch.–Asp.	C	Ch	Asp.	Ch.–Asp.	C	Ch	Asp.	Ch.–Asp.	C	Ch	Asp.	Ch.–Asp.	C	Ch	Asp.	Ch–Asp.
PAO1	7	4	3	3	5	5	2	2	0.21 (+2)	0.012 (0)	0.018 (0)	0.01 (0)	19.88	7.68	11.41	6.196	(0)	(0)	(0)	(0)
					Clinical isolates															
ps34	6	6	4	3	4	4	3	2	0.22 (+2)	0.011 (0)	0.013 (0)	0.009 (0)	25.69	8.51	12.90	6.393	(+2)	(+1)	(+1)	(0)
ps36	6	6	2	2	6	3	2	2	0.24 (+2)	0.025 (0)	0.025 (0)	0.012 (0)	8.826	5.83	7.68	5.852	(+2)	(0)	(0)	(0)
ps37	7	7	3	3	5	3	2	2	0.26 (+2)	0.01 (0)	0.009 (0)	0.008 (0)	16.48	4.06	10.82	5.166	(+2)	(0)	(+1)	(0)
ps45	5	3	3	3	7	3	2	2	0.2 (+2)	0.012 (0)	0.01 (0)	0.013 (0)	15.71	4.56	14.50	5.387	(+2)	(+1)	(+1)	(+1)
ps47	5	5	3	3	6	3	3	3	0.23 (+2)	0.014 (0)	0.01 (0)	0.009 (0)	31.22	6.51	17.17	7.336	(+2)	(+1)	(+1)	(+1)
ps62	6	6	3	3	7	4	2	2	0.24 (+2)	0.016 (0)	0.01 (0)	0.01 (0)	11.94	6.24	9.679	6.820	(+2)	(0)	(0)	(0)
ps63	5	5	3	3	5	5	2	2	0.44 (+3)	0.044 (0)	0.011 (0)	0.011 (0)	28.61	7.57	13.21	4.228	(+2)	(+1)	(+1)	(+1)
ps88	6	5	3	3	5	4	3	3	0.18 (+1)	0.011 (0)	0.009 (0)	0.01 (0)	23.74	3.82	11.11	7.323	(+2)	(0)	(+1)	(+1)
ps99	6	4	4	3	6	5	5	2	0.18 (+1)	0.097 (0)	0.012 (0)	0.011 (0)	32.15	6.04	12.85	8.954	(+2)	(0)	(+1)	(0)
ps100	7	4	3	3	5	4	2	2	0.21 (+2)	0.01 (0)	0.009 (0)	0.01 (0)	25.23	5.70	12.46	6.075	(+2)	(0)	(+1)	(0)
Mean (*n* = 10)	5.9	5.1	3.1	2.9	5.6	3.8	2.6	2.3	0.240	0.025	0.012	0.010	21.963	5.89	12.24	6.353	2	0.4	0.7	0.4
±SD	0.7	1.2	0.6	0.3	1	0.8	1	0.5	0.075	0.027	0.005	0.001	8.192	1.47	2.623	1.337	0	5	0.5	0.5

C: control without aspirin or chitosan–aspirin, Ch: chitosan, Asp.: aspirin, Ch.–Asp.: chitosan–aspirin, Å: absorbance. The strength of biofilm production was graded on a scale of 0 to 3 based on the OD, as follows: OD ≤ 0.09 = none (0); 0.09 < OD ≤ 0.18 = weak (1+); 0.18 < OD ≤ 0.36 = moderate (2+); 0.36 < OD = strong (3+). The QS capacity was scored from 0 to +2 based on the intensities of AHL processing, as follows: zone diameter (mm) = none (0); mm ≤ 2.5 = moderate; 2.5 < mm = strong.

**Table 3:** Measurement of *LasI* and *RhlI* expression in *P. aeruginosa* after treatment with chitosan–aspirin.

	Cycle Threshold (CT) of *LasI*			Inhibition of *LasI*		Cycle Threshold (CT) of *RhlI*			Inhibition of *RhlI*	
	C	Ch.	Ch.–Asp.	Ch.	Ch.–Asp.	C	Ch.	Ch.–Asp.	Ch.	Ch.–Asp.
PAO1	21.7	22.8	23.5	1.39	7.12	16.2	17.8	18.9	2.09	0.92
Clinical isolates (*n* = 10)										
ps34	18.7	23.0	21.0	84.5	1.0	13.7	23.0	21.0	2.70 × 10^3^	2.28 × 10^2^
ps36	16.5	20.8	18.0	1.11 × 10^2^	1.65 × 10^2^	15.2	17.5	19.4	25.6	6.6
ps37	13.0	19.3	18.9	5.26 × 10^2^	1.40 × 10^2^	16.4	22.6	39.0	4.71 × 10^2^	1.83 × 10^7^
ps45	20.6	21.6	24.5	36.0	1.86 × 10^2^	19.4	21.3	35.1	68.17	5.11 × 10^5^
ps47	24.8	26.8	26.8	7.76	5.87	20.8	23.5	24.1	12.99	4.51 × 10^2^
ps62	19.9	21.4	23.0	1.64	3.57 × 10^6^	19.0	20.2	20.3	1.40	1.73
ps63	16.2	18.1	37.0	16.73	4.34 × 10^3^	16.2	18.1	33.6	16.73	3.41 × 10^5^
ps88	11.7	27.5	23.9	9.357 × 10^4^	5.91 × 10^3^	10.4	23.9	29.4	1.85 × 10^4^	4.8 × 10^5^
ps99	20.4	32.0	32.2	2.70 × 10^3^	3.95 × 10^9^	14.1	16.3	16.7	3.802	9.53
ps100	4.6	31.7	37.0	9.46 × 10^7^	0.47	6.4	16.1	21.9	5.77 × 10^2^	3.22 × 10^4^
Median	17.6	22.3	24.23	98.08	1.76 × 10^2^	15.8	20.7	23.05	46.91	1.63 × 10^4^

## Data Availability

All data are included in this published article. The datasets generated during the current study are available at https://www.ncbi.nlm.nih.gov/genbank/, www.uniprot.org, www.swissmodel.expasy.org/, and https://3dsbiovia.com/resource-center/, all accessed on 2 March 2024.
